# Interaction between Thymidylate Synthase and Its Cognate mRNA in Zebrafish Embryos

**DOI:** 10.1371/journal.pone.0010618

**Published:** 2010-05-12

**Authors:** Yuyan Zhang, Shaoli Yang, Ming Liu, Chunxia Song, Ning Wu, Peixue Ling, Edward Chu, Xiukun Lin

**Affiliations:** 1 Institute of Oceanology, Chinese Academy of Science, Qingdao, People's Republic of China; 2 Yale Cancer Center, Yale University School of Medicine, New Haven, Connecticut, United States of America; 3 Graduate School of Chinese Academy of Sciences, Beijing, People's Republic of China; 4 Institute of Biopharmaceuticals of Shandong Province, Jinan, People's Republic of China; Dr. Margarete Fischer-Bosch Institute of Clinical Pharmacology, Germany

## Abstract

Thymidylate synthase (TS), which catalyzes the de novo synthesis of dUMP, is an important target for cancer therapy. In this report, the effects of 5-fluorouracil (5-FU) and ZD1694 on the regulation of TS gene expression were evaluated in zebrafish embryos. Our results revealed that the expression of TS was increased by about six-fold when embryos were treated with 1.0 µM 5-FU and there was a greater than 10-fold increase in the TS protein level after treatment with 0.4 µM ZD1694. Northern blot analysis confirmed that expression of TS mRNA was identical in treated or untreated embryos. Gel shift and immunoprecipitation assays revealed that zebrafish TS was specifically bound with its cognate mRNA in vitro and in vivo. We identified a 20 nt RNA sequence, TS:N20, localized to the 5′-UTR of TS mRNA, which corresponded to nt 13–32; TS:N20 bound to the TS protein with an affinity similar to that of the full-length TS mRNA. The MFold program predicted that TS:N20 formed a stable stem-loop structure similar to that of the cis-acting element found in human TS mRNA. Variant RNAs with either a deletion or mutation in the core motif of TS:N20 were unable to bind to the TS protein. In vitro translation experiments, using the rabbit lysate system, confirmed that zebrafish TS mRNA translation was significantly repressed when an excess amount of TS protein was included in the system. Additionally, a TS stability experiment confirmed that treatment of zebrafish embryos with 5-FU could increase the TS stability significantly, and the half life of TS protein was about 2.7 times longer than in untreated embryos. Our study revealed a structural requirement for the interaction of TS RNA with TS protein. These findings also demonstrated that the increase in TS protein induced by 5-FU occurs at the post-transcriptional level and that increased stability and translation efficiency both contributed to the increase in TS protein levels induced by TS inhibitors.

## Introduction

Thymidylate synthase (TS) is a folate-dependent enzyme that catalyzes the reductive methylation of dUMP to dTMP using 5, 10-methylenetetrahydrofolate as a one-carbon donor. This enzyme has been an important target for cancer chemotherapy for several decades because TS represents the sole de novo source of thymidylate (dTTP), which is essential for DNA replication and repair [1], [2]. A number of studies using cultured cell lines, tumor models and clinical specimens have shown that TS inhibitors induce about a 2- to 4-fold increase in TS levels [3], [4]. Such an induction has been viewed as a potential barrier to successful therapeutic outcomes because the response to TS-directed chemotherapy is dependent on the enzyme concentration [5]. As a result, extensive studies have been performed to elucidate the mechanisms of inhibitor-mediated induction of TS and to develop strategies to ameliorate the unwanted effects. Our previous study confirmed that TS, in addition to its role in enzymatic catalysis, also functions as an RNA binding protein [6], [7]. Specifically, translation of human TS mRNA is negatively regulated by direct binding of TS to two different *cis*-acting elements in its cognate mRNA. The first element is a 30-nt sequence found within the 5′-untranslated region (5′-UTR) that includes the translational start site in a stable stem-loop structure [8]. The second binding site is a 70-nt sequence corresponding to the nucleotides 480–550 [9]. When 5-fluorouracil (5-FU) or other TS inhibitors bind to TS, the complex cannot interact with its cognate mRNA, which results in increased TS protein expression [10].

There is additional evidence demonstrating that the fluoropyrimidine-mediated increase in TS protein expression is also due to enhanced stability; treatment of human colon cancer HCT-15 cells with 5-FU, or other TS inhibitors, resulted in enhanced TS protein stability. The half-life of TS in 5-FU-treated cells was increased to around 25 h, compared to about 7 h for untreated cells [11]. Further study suggested that 5-FU may interact with the inactive form of TS, increasing its stability by changing the molecular ternary conformation [12]. However, all of the above studies were performed in cancer or normal cell lines. Further study is essential to elucidate the mechanism of 5-FU-induced over-expression in animal models.

The functionality of RNA depends not only on the primary sequence but also on secondary folding. Work on RNA motif discovery is relevant to better understand the metabolism of RNA, which essentially relies on the interaction of transcripts with proteins [13]. A web server for RNA data analysis, which has significant capabilities for predicting the secondary structure of RNA, has been reported by Khaladkar et al [14]. However, much work remains to be done to determine the molecular mechanisms underlying the structural requirements for protein-RNA interaction [15]. Our previous studies demonstrated that an intact stem-loop structure in the *cis*-acting element, localized up-stream of human TS mRNA, is essential for TS mRNA-protein interaction. However, the two *cis*-acting elements in human TS mRNA do not display any homology in their primary sequences required for human TS protein binding [8]. Deletion or mutation of key nucleotides in the *cis*-acting element of human TS mRNA results in loss of TS protein binding activity [16]. Further study is needed to address whether there is similarity in the secondary structure of the *cis*-acting elements in TS mRNA among different species.

The zebrafish model system is excellent for genetic, embryological, developmental and cell biological studies [17]. The externally developing embryos are transparent, allowing for visualization of organ systems. Zebrafish embryos exhibit unique characteristics including ease of maintenance and drug administration, a short reproductive cycle and transparency that permit visual assessment of developing cells and organs. In recent years, the zebrafish embryo has become an important vertebrate model for assessing drug effects [18]. A previous study in our laboratory demonstrated that zebrafish TS is a dimer with a molecular weight of 72 kDa. Sequence analysis indicated that there is high similarity (76%) between zebrafish and human TS, with the same folate binding site and 93% similarity to the dUMP binding site [19]. However, little is known regarding TS regulation in response to 5-FU in zebrafish embryos. Additionally, in order to develop zebrafish as a whole-animal model for screening novel TS inhibitors, a systematic study is a prerequisite for the elucidation of the TS regulatory mechanism in zebrafish.

In the present report, using both in vitro and in vivo model systems, we have demonstrated that zebrafish TS protein binds to its own cognate mRNA, and the translational efficiency is significantly suppressed by an excessive amount of TS protein. Gel shift experiments and competition analyses revealed that a 20-nt sequence in the 5′-UTR of TS RNA is a critical site for TS RNA–protein interaction. Furthermore, Western blot analysis confirmed that TS stability was also significantly increased in response to treatment with 5-FU.

## Results

**Figure 1:**
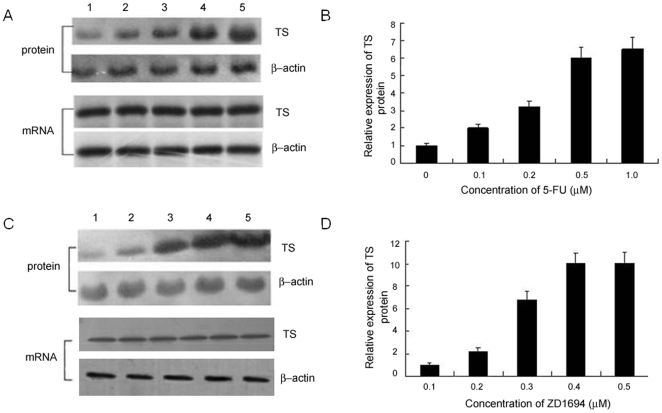
Expression profile of TS mRNA and protein in zebrafish embryos treated with 5-FU or ZD1694. Zebrafish embryos were treated with various concentrations of 5-FU or ZD1694 for 24 h and the expression levels of TS mRNA and protein were evaluated. The TS mRNA and protein level after 5-FU (*A*, *B*) or ZD1694 (*C*, *D*) treatment is shown. *A*, Zebrafish embryos were untreated (lane 1) or exposed to 0.1 µM (lane 2), 0.2 µM (lane 3), 0.5 µM (lane 4) or 1.0 µM 5-FU (lane 5) for 24 h, and TS mRNA and protein expression was determined using Northern and Western blotting analysis respectively. *C*, TS mRNA and protein level in untreated embryos (lane 1) or embryos treated with 0.1 µM (lane 2), 0.2 µM (lane 3), 0.3 µM (lane 4) and 0.4 µM (lane 5) ZD1694, respectively. *B* and *D*, The quantification results of protein expression, which represent the protein expression level after 5-FU or ZD1694 treatment, respectively, is shown. The TS expression level in untreated embryos was defined as 1.0.

It is well documented that TS inhibitors such as 5-FU and ZD1694 can promote a 2–4-fold increase in the TS enzyme level in cultured cell lines, tumor models and clinical specimens [3], [4]. To determine if the treatment of zebrafish embryos with 5-FU or ZD1694 would also result in the over-expression of TS, Western immunoblot analysis was performed to evaluate the TS expression levels in untreated and 5-FU- or ZD1694-treated embryos. As shown in figure 1, both 5-FU and ZD1694 significantly increased TS expression in zebrafish embryos. At a concentration of 0.1–1.0 µM 5-FU, TS expression was increased in a dose-dependent manner (Fig. 1A, lane 1–5). The highest induction of TS expression, which was about a 6.5-fold increase over untreated embryos, was found in those treated with 1.0 µM 5-FU (Fig. 1 A, B). Increasing 5-FU concentration to 2 µM did not result in an additional enhancement in TS expression (data not shown). In embryos treated with ZD1694, TS expression was more potently increased; treatment with 0.4 µM ZD1694 resulted in 10 times more TS expression compared to untreated embryos (Fig. 1C, D). Of note, the expression of β-actin, remained unchanged in both untreated and treated embryos. Northern blot analyses were also performed to determine changes in the TS mRNA level in response to 5-FU and ZD1694 treatment. Treatment of zebrafish embryos with 0.1, 0.2, 0.5 or 1.0 µM 5-FU for 24 h did not result in any alteration of the TS mRNA level (Fig. 1A, panel 3). Similarly, the mRNA level was also unaffected following treatment with various concentrations of ZD1694 (Fig. 1C, panel 3). These findings suggested that the induction of TS protein in zebrafish after drug treatment is dose-dependent and occurs at the post-transcriptional level.

**Figure 2:**
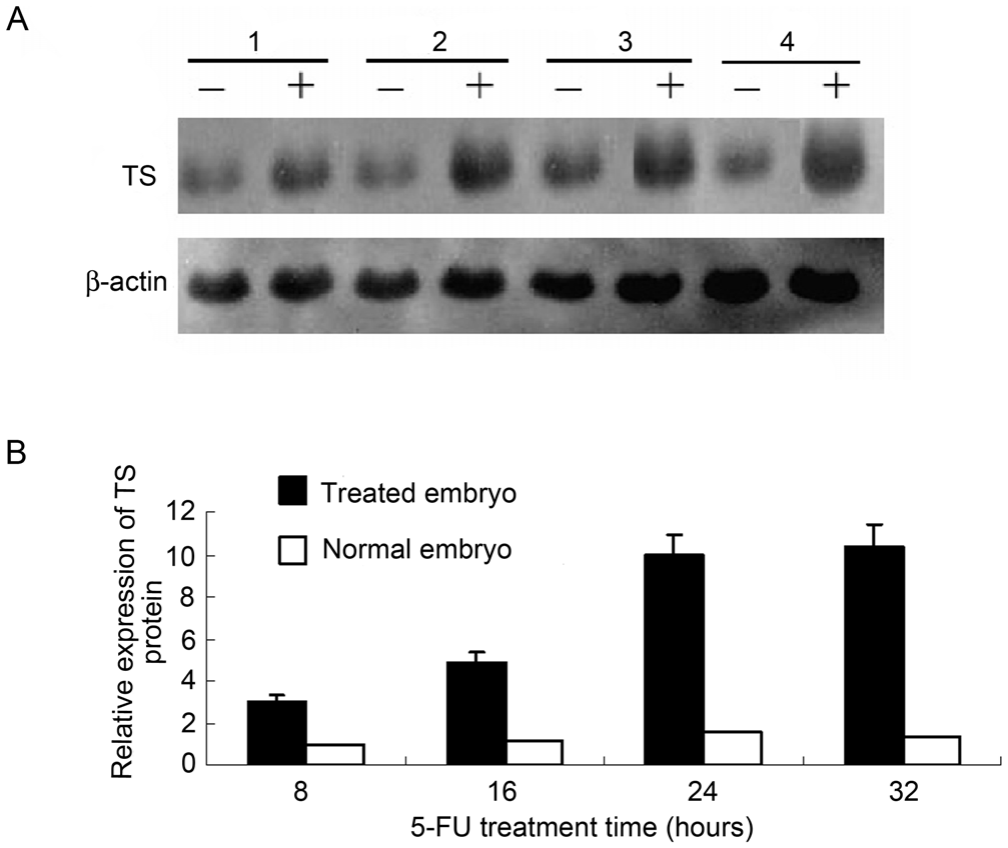
Time-dependent effect of 5-FU on TS expression. Zebrafish embryos (n = 100) were untreated or treated with varying concentrations of 5-FU. A. Western immunoblot analyses were performed to evaluate TS levels in untreated zebrafish embryos or in embryos treated with 1.0 µM 5-FU for 8 h (lane 1), 16 h (lane 2), 24 h (lane 3) and 32 h (lane 4). β-actin was used as internal control. B. The quantification results of protein expression. The expression level of TS in untreated embryos at 8 h post-fertilization (hpf) was defined as 1.0. Each experiment was performed more than three times.

To more precisely identify the underlying molecular events of the regulation of TS gene expression, the level of TS protein was determined in response to 5-FU drug treatment for varying amounts of time. Densitometry analysis of the Western immunoblot experiments demonstrated a 3.0-, 4.1-, 6.2- and 7.4-fold increase (Fig. 2B) in TS protein when zebrafish embryos were treated with 1.0 µM 5-FU for 8 (Fig. 2A, lane 1), 16 (Fig. 2A, lane 2), 24 (Fig. 2A, lane 3) or 32 h (Fig. 2A, lane 4), respectively. Furthermore, TS expression in untreated embryos remained mostly unchanged from 8 to 32 h (Fig. 2A, lane 1–4). These results suggest that 5-FU induced the expression of TS in zebrafish in a time-dependent manner.

**Figure 3:**
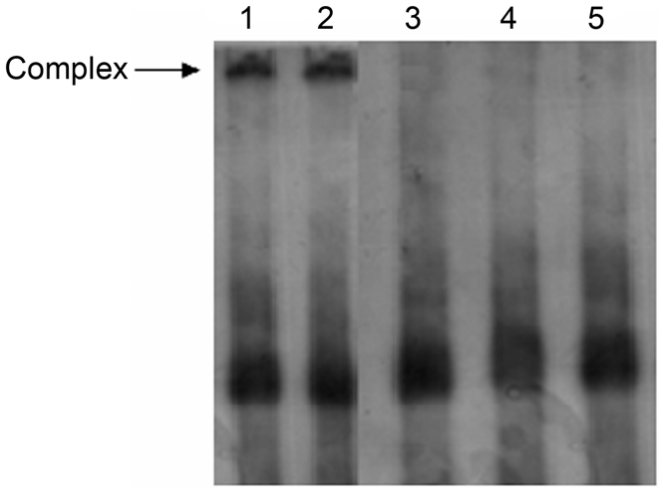
Specific interaction between zebrafish TS and its cognate mRNA in vitro. A gel shift experiment was performed using ^32^P-labeled full-length TS mRNA as the probe. Zebrafish TS protein (100 ng) was incubated with ^32^P-labeled full-length TS mRNA (lane 1, arrow). One hundred-fold molar excess luciferase mRNA did not affect the interaction between TS protein and mRNA (lane 2). In lane 3, 100-fold molar excess of unlabeled TS mRNA was included in the incubation buffer. Using bovine serum albumin in place of the TS protein did not result in any specific interaction (lane 4). Lane 5 contained 100 ng ^32^P-labeled zebrafish TS mRNA only.

To determine if TS could bind to its cognate mRNA in vitro, we performed an electrophoretic mobility shift assay (EMSA) with TS recombinant protein and ^32^P-labeled full-length TS mRNA. A TS mRNA probe was incubated with TS protein and analyzed using a non-denaturing 5% acrylamide gel. The results demonstrated the presence of a specific RNA-TS complex (Fig. 3, lane 1). To determine the specificity of the TS–mRNA interaction, a 100-fold excess of luciferase mRNA was included in the reaction mixture, and the results demonstrated that luciferase mRNA did not affect the binding of TS protein to its own mRNA (Fig. 3, lane 2). In contrast, no interaction was found when an excess amount of TS mRNA was added to the reaction mixture (Fig. 3, lane 3). Replacing TS protein with BSA also did not lead to a bound complex (Fig. 3, lane 4). These results demonstrated that the TS protein could bind specifically to its cognate mRNA in vitro.

In order to more precisely localize the TS binding site in the full-length TS mRNA, we initially performed a series of RNA competition gel mobility-shift experiments. Several TS RNA sequences from zebrafish TS mRNA were synthesized in vitro and used as unlabeled RNA competitors in gel mobility-shift assays to determine the relative binding affinity (IC_50_) of zebrafish TS protein for each sequence (Table 1). Full-length TS mRNA effectively competed for TS protein binding (Table 1, IC_50_ = 0.50 nM), while the luciferase mRNA had a 1000-fold lower protein binding affinity than the TS full-length mRNA (Table 1). TS mRNA sequences, including TS 1–580, TS 1–290, TS 1–145, TS 1–72 and TS 1–36 displayed similar TS protein binding activity as the full-length TS mRNA in the competition assay (Table 1). However, the TS mRNA sequences TS 581–1163, TS 291–580, TS 146–290, TS 73–145 and TS 37–74 were unable to compete for TS protein binding (Table 1). The results from these competition experiments suggested the presence of a TS binding site within the first 37 nt of the TS mRNA.

**Table 1:** Relative binding affinity of TS RNA constructs.

Constructs	Sequences	IC_50_, nM
Full-length TSTS1–1163		0.50±0.06
TS1–580		0.48±0.08
TS1–290		0.48±0.07
TS1–145		0.52±0.08
TS1–72		0.65±0.11
TS1–36		0.60±0.14
TS:N20(TS13–32)	5′-CUCUUGUUGUGCUGCAGGAU-3′	0.62±0.20
Δ20-TS RNA	5′-CUCUUGUUGUGCAGGAU-3′	>1000
Δ20-18-TS RNA	5′-CUCUU**CA**UGUGCUGCAGGAU-3′	>1000
Δ20-22-TS RNA	5′-CUCUUGUUGU**AAA**GCAGGAU-3′	>1000
TS581–1163		>1000
TS291–580		>1000
TS146–290		>1000
TS73–145		>1000
TS37–74		>1000
Luciferase RNA		>1000

Binding of human TS protein to various RNA sequences was determined as described in the Materials and Methods. The nucleotides in bold represent point mutations. Each value represents the mean±SE of between three and five experiments.

**Figure 4:**
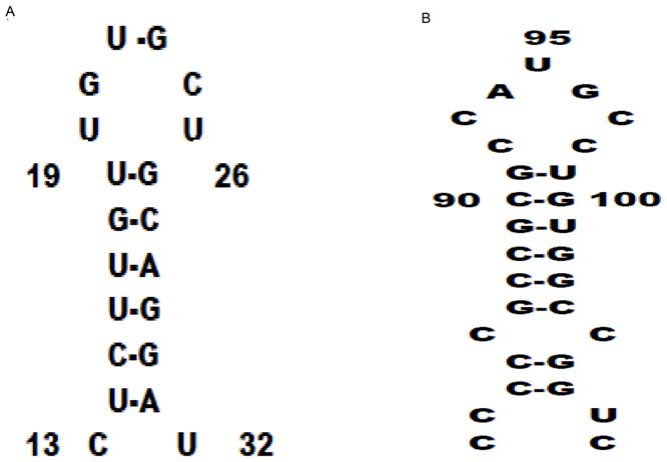
Secondary structure of TS:N20. *A*, Secondary structure for the zebrafish TS RNA sequence corresponding to nt 13–32, TS:N20, which was predicted with the RNA MFold program, generating a stem-loop secondary structure. *B*, The secondary structure of the *cis*-acting element localized in the upstream region of human TS mRNA [8], which binds specifically with human TS.

Previous studies have demonstrated that a stem-loop secondary structure is present in the upstream region of human TS mRNA, which included AUG site, that plays key roles in RNA and human TS protein interaction [8], [16]. In the present study, the first 37 nt in the TS RNA sequence were subjected to MFOLD analysis (http://mfold.bioinfo.rpi.edu/cgi-bin/rna-form1.cgi) and the predicted stable stem-loop structure was found in nt13–32 in TS mRNA, designated TS:N20 (Fig. 4A). To more accurately define the sequence and/or structural requirements for the RNA–protein complex formation within this specific region, we synthesized the 20-nt RNA construct (nt 13–32) found in the 5′UTR of the TS RNA sequence. TS:N20 competed for TS protein binding with activity similar to the full-length TS RNA (Table 1, IC_50_ = 0.62 nM).

**Figure 5:**
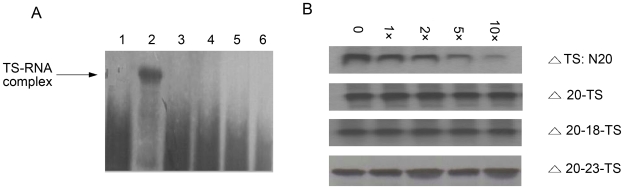
Interaction of TS:N20 with zebrafish TS protein in vitro. *A*, Gel mobility shift analysis of TS:N20 interaction with TS protein. A 20-nt TS RNA, which included nt 13–32, was synthesized and radio-labeled with ^32^P. ^32^P-labeled-TS:N20 RNA was incubated in the absence (lane 1) or presence of 100 ng (lane 2) of pure recombinant zebrafish TS protein. A ^32^P-labeled 17-nt TS encompassing nt 13–32 with a deletion of the GCU sequence, Δ20-TS RNA, was incubated in the presence of 100 ng of zebrafish TS protein (lane 3). Lanes 4 and 5 represent the other two mutation variants, Δ20–18-TS RNA and Δ20–23-TS RNA, respectively, incubated with 100 ng of pure TS protein. TS:N25 RNA was incubated with 100 ng TS protein in the presence of 100 ng unlabeled full-length TS mRNA (lane 6). Samples were resolved on a non-denaturing 5% acrylamide gel and visualized by autoradiography. The specific RNA–protein complex is indicated (arrow). *B*, Competition analysis using TS oligomers with TS protein. ^32^P-labeled full-length TS RNA (10^5^ cpm) was incubated with 200 ng of pure zebrafish recombinant TS protein in the presence of 0–10-fold molar excess competitor, TS:N20 RNA (panel 1), Δ20-TS RNA (panel 2), Δ20–18-TS RNA (panel 3) and Δ20–23-TS RNA (panel 4). The RNA–protein complex was resolved on a non-denaturing 5% acrylamide gel and visualized by autoradiography.

An RNA gel mobility shift assay was used to determine structural requirements for the TS:N20–TS protein interaction. ^32^P-labled TS:N20 RNA could form an RNA–protein complex in the presence of 100 ng pure recombinant TS protein (Fig. 5A, lane 2). In contrast, deleting of the UGC sequence in the loop structure of theTS:N20, the mutant Δ20-TS resulted in complete loss of this interaction (Fig. 5A, lane 3). To provide further support for the importance for maitain an intact stem-loop structure, two other TS RNA variants were synthesized: Δ20–18-TS RNA, which contained a two base substitution, GU→CA, at nt 18 and 19, and Δ20–23-TS RNA, which contained base substitutions, GCU→AAA, at nt 23–25. These two TS RNA variants were unable to form a TS–RNA complex with the zebrafish TS protein (Fig. 5A, lanes 4 and 5). Additionally, when 100 ng full-length TS mRNA was included in the reaction mixture, the interaction of TS:N20 with TS protein was abolished completely (Fig. 5A, lane 6). To more precisely measure the ability of these three RNAs to interact with TS protein, we determined their relative binding affinity for TS protein compared to full-length RNA. TS:N20 bound TS protein with a relative affinity (Table 1, IC_50_ = 0.62 nM; Fig. 5B, panel 1) similar to the full-length RNA. The three RNA variants, Δ20-TS RNA, Δ20–18-TS RNA and Δ20–23-TS RNA, were unable to compete with ^32^P-labeled full-length TS RNA for TS protein binding, even at a high concentration (Fig. 5B, panel 2–4). The MFold program was used to predict the stability of the secondary structure of TS:N20 and its mutant constructs (Table 2). Like TS:N20, both Δ20–18-TS and Δ20–23-TS RNA could form a stable stem-loop structure. However, the loop of TS:N20 consisted of six nucleotides, while the loop in Δ20–18-TS and Δ20–23-TS RNA contained only five and four nucleotides, respectively. These findings suggested that TS:N20 localized to the 5′UTR of the TS RNA sequence is a critical site for TS protein binding, and that some specific nucleotides and the loop size of the secondary structure formed by the 20-nt sequence play a key role in RNA–TS protein interaction.

**Table 2:** Stability of TS:N20 and its mutant constructs.

Constructs	Sequences	dG (kcal/mol)
TS:N20(TS13–32)	5′-CUCUUGUUGUGCUGCAGGAU-3′	−5.3
Δ20-TS RNA	5′-CUCUUGUUGUGCAGGAU-3′	−4
Δ20–18-TS RNA	5′-CUCUU**CA**UGUGCUGCAGGAU-3′	−0.7
Δ20–23-TS RNA	5′-CUCUUGUUGU**AAA**GCAGGAU-3′	−6.3

MFold Program was used to predict the stability of the secondary structure of TS:N20 RNA and the mutant constructs. The nucleotides in bold represent point mutations.

**Figure 6:**
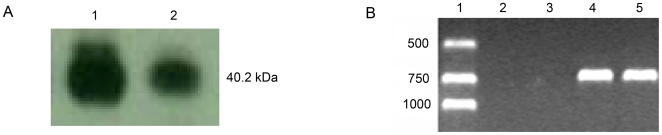
Zebrafish TS protein interacted with its own mRNA in vivo. *A*, Western blot analysis was performed with purified recombinant zebrafish TS (lane 1) or protein from the immunoprecipitation complex (lane 2) and detected with human TS106 antibody. *B*, RT-PCR products using the RNA extracted from immunoprecipitation complex as the template. TS mRNA from nt 340–1040 was amplified by RT-PCR using TS-specific primers (lanes 4 and 5). RNA and protein complex was precipitated without TS antibody and RT-PCR was performed (lane 2). Lane 3 indicates the RT-PCR results precipitating the complex with unrelated polyclonal antibody, CHX10-like zebrafish antibody (GenWay Biotech, CA). Lane 1 represents the DNA marker of molecular weight.

To further assess the *in vivo* binding activity of TS protein, zebrafish embryos were homogenized and the TS–mRNA complex was immunoprecipitated using a TS monoclonal antibody. Western blot analysis and RT-PCR experiments were performed to determine if the precipitated complex included TS mRNA and TS protein. The results demonstrated that TS protein could be co-precipitated in the presence of the monoclonal antibody, TS106 (Fig. 6A, lane 2). TS mRNA from nt 340–1040 was also amplified by RT-PCR using TS-specific primers (Fig. 6B, lane 4 and 5). In contrast, no specific interaction was detected without the TS antibody (Fig. 6B, lane 2). Additionally, when an unrelated monoclonal antibody was used, no amplified RNA was detected (Fig. 6B, lane 3). These results confirmed that TS interacted with its own RNA in vivo specifically.

**Figure 7:**
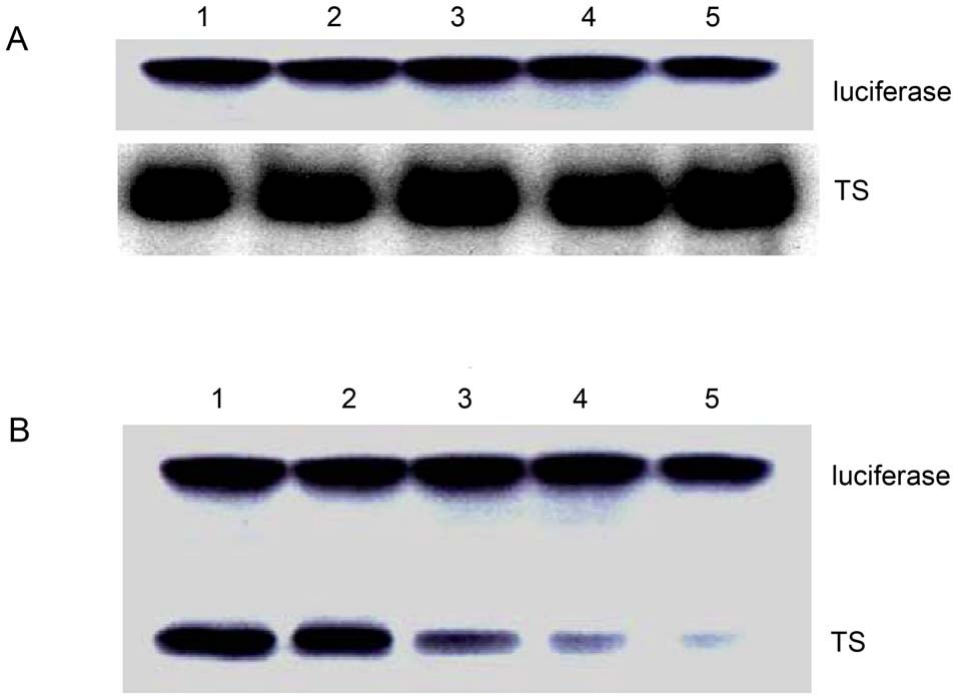
TS protein inhibited in vitro translation of zebrafish TS mRNA. A rabbit reticulocyte lysate in vitro translation system was used to determine the interaction between mRNA and TS protein. Incubation of zebrafish TS mRNA with the reticulocyte lysate yielded a protein product with a molecular mass of approximately 35 kDa. *A*, Zebrafish TS mRNA was incubated in the rabbit reticulocyte lysate system without (lane 1) or with 100 ng of BSA (lane 2), luciferase (lane 3), human p53 protein (lane 4) or alpha-chymotrypsin (lane 5). *B*, Excess TS protein was included in the reaction mixture. Zebrafish TS mRNA was incubated in the translation system without (lane 1) or with 1 ng (lane 2), 10 ng (lane 3), 100 ng (lane 4) or 200 ng (lane 5) TS protein.

To determine the biological relevance of the TS protein–mRNA interaction, a rabbit reticulocyte lysate in vitro translation system was used. Incubation of zebrafish TS mRNA with the reticulocyte lysate yielded a protein product with a molecular mass of approximately 35 kDa (Fig. 7A, lane 1). To determine if the translation of TS was affected by the presence of other proteins, BSA (Fig. 7A, lane 2), luciferase (Fig. 7A, lane 3), human p53 protein (Fig. 7A, lane 4) or alpha-chymotrypsin (Fig. 7A, lane 5) were included in the translation mixture. Our results showed that unrelated proteins did not affect TS translation (Fig. 7A, lane 1–5). In contrast, when an excess amount of zebrafish TS protein was added to the reaction mixture, the translation of TS mRNA was significantly inhibited (Fig. 7B, lane 1–5). The formation of TS in the translation system was negatively dose-dependent on the amount of TS added to the translation system (Fig. 7B, lane 1–5).

**Figure 8:**
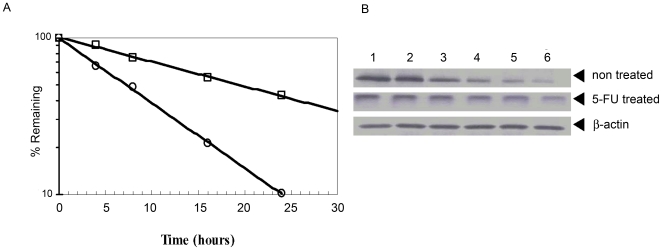
Effect of 5-FU on the stability of TS protein in zebrafish embryos. Healthy embryos at the 1–2 cell stage were treated with 10 µg/mL cycloheximide and 0.5 µg/mL 5-FU and incubated for another 4, 8, 16, 24 and 36 h. Embryos (n = 100) that were untreated or treated with cycloheximide and 5-FU were collected and the total protein was extracted for Western blot analysis as described in the Materials and Methods section. *A*, TS level in the zebrafish embryo at various time points after 5-FU treatment. TS level in the absence (circles) or presence of 5-FU (box) was analyzed by Western blot and quantified using a Hewlett Packard ScanJet 4P and NIH image 1.51 software. *B*, Western blot analysis showing the amount of TS at specific times after treatment with cycloheximide. Zebrafish TS embryos were left untreated (panel 1) or treated with 5-FU (panel 2) for 2 h, and 10 µg/mL cycloheximide was added to incubated for another 0 (lane 1), 4 (lane 2), 8 (lane 3), 16 (lane 4), 24 (lane 5) and 36 h (lane 6). Western blots were performed as described in the Materials and Methods section.

Studies by Berger et al. revealed that treatment of cancer cells with 5-FU resulted in increased stability of the TS protein [20]. In order to determine if treatment with 5-FU affected TS stability in zebrafish embryos, we examined the TS remained after the embryos were treated with cycloheximide and 5-FU at different times. As shown in Fig. 8B, 5-FU treatment of the embryos significantly increased TS stability. Compared with the untreated embryos, the half-life of TS increased by about 2.7 times; the half-life of zebrafish TS increased from 7.2 h to around 19.5 h after 5-FU treatment (Fig. 8A). These results suggested that treatment of embryos with 5-FU could strongly enhance the stability of TS.

## Discussion

TS catalyzes the conversion of dUMP to dTMP and is therefore indispensable for DNA replication in actively dividing cells. The enzyme is a critical target for chemotherapeutic agents such as fluoropyrimidines (e.g., 5-FU and 5-fluoro-2′-deoxyuridine) and folic acid analogues (e.g., raltitrexed, LY231514, ZD9331 and BW1843U89) [1]. In this study, we demonstrated for the first time that, after short-term exposure of zebrafish to 5-FU or ZD1694, TS enzyme levels were elevated significantly. The enhancement of TS expression induced by 5-FU is attributable to increased translational efficiency as well as enhanced TS protein stability.

In our previous study, we found that human TS could bind to its cognate mRNA and repress the translation of TS mRNA. Two binding sites have been identified in human TS mRNA. One binding site is located in the 5′ region, encompassing a 30-nt sequence that includes the translation start site, forming a stable stem-loop structure [8]. The second binding site is a 70-nt sequence in the protein-coding region that corresponds to nucleotides 480–550 [16]. No similar sequences were found in the corresponding regions of the zebrafish TS mRNA [19]. In the present study, using gel mobility-shift analysis as well as competition experiments, we have identified a *cis*-acting element in the 5′UTR of zebrafish TS mRNA. The RNA MFold program predicted that this element, TS:N20, adopts a stable stem-loop structure. Moreover, the three nucleotide sequence, UGC, which is in the loop motif, and an intact stem-loop structure formed by six-nucleotides are required for zebrafish TS protein–RNA binding. Our previous studies demonstrated that a *cis*-acting element that includes AUG localized upstream of human TS mRNA, could form a stem-loop structure [6] that binds specifically to human TS protein. Compared with the *cis*-acting element in the upstream of human TS RNA (Fig. 4B), there is high similarity in the secondary structure between the human and zebrafish *cis*-acting elements. First, the core motif of both cis-elements is made up of a stem consisting of a hexanucleotide and a loop structure with six to seven nucleotides. Second, a non-Watson-Crick GU base pairing is essential for maintaining the intact stem-loop structure that allows for optimal interaction with the TS protein. A precedent of the structural requirement study demonstrated that maintaining an intact stem-loop structure is critical for protein interaction with RNA. A stem-loop structure localized to the 3′UTR of the β-subunit of the F0F1-ATPase mRNA is required for the formation of an RNA–protein complex [21]. Iron regulatory protein 1 (IRP1) binds iron-responsive elements (IREs) in the 5′UTR of mRNAs to repress translation or degradation. Further studies revealed that the extended, L-shaped IRP1 molecule bound to the IRE stem-loop through interaction at two sites separated by approximately 30 Å, each involving about a dozen protein–RNA bonds [22]. Our present study provides additional evidence that maintaining some precise secondary structure of *cis*-acting elements is necessary for RNA–protein complex formation. We are now trying to dissect the exact interaction between the TS protein and the cis-element.

The redox state of TS plays a key role in the interaction between human TS and mRNA. In the presence of reducing agents, such as 2-mercaptoethanol and/or dithiothreitol, the RNA binding activity of TS is significantly enhanced. In contrast, treatment with oxidizing agents, such as diamide or N-ethylmaleimide, significantly inhibits RNA binding activity [23]. Further study in our laboratory confirmed that one of the amino acid residues of human TS is crucial for TS–mRNA interaction and a cysteine to alanine mutation at cysteine residue 180 completely eliminated RNA binding activity. Mutations of other cysteines did not impact RNA binding [24]. There are six cysteine residues in the zebrafish TS molecule [19]; we found that oxidizing agents like diamide also inhibited the interaction between zebrafish TS and mRNA (data not shown). We are currently examining which of the cysteine residues are essential for RNA binding.

Previous studies have shown that human TS could also bind with p53 and c-myc mRNA, repressing their translation in human colon cancer cells [6], [25]. Cancer cells that overexpress TS and have suppressed levels of p53 are significantly impaired in their ability to arrest in G1 phase in response to exposure to a DNA-damaging agent such as gamma-irradiation [25]. Human TS could bind with the C-terminal coding region of c-myc mRNA and repressed its translation [26]. Considering the high similarity between human and zebrafish TS, it is conceivable that zebrafish TS may interact with c-myc and p53 in zebrafish embryos. Studies are in progress in our laboratory to address if there are interaction among TS and c-myc and/or p53 mRNA and if the interaction affects the embronic development. Additionally, like in human cancer cells, our initial studies confirmed that the metabolite of 5-FU, FdUMP could also increase the expression of TS in zebrafish embryos, and treatment of the embryos with FdUMP also enhanced TS stability significantly. Treatment of embryos with FdUMP (0.5 µM, Sigma, St. Louis, MO) increased TS level by about 7-fold (data not shown), suggesting a similar mechanism in this species as what is seen in humans.

Studies by other investigators have also found that the elevated expression of TS after exposure to TS inhibitors was attributable to increased protein stability [24]. The half-life of TS was significantly increased in 5-FU-treated HCT-15 colon cancer cells [11]. In the present study, we found that treatment of zebrafish embryos with 5-FU increased the stability of TS protein significantly and the half-life of TS in 5-FU-treated embryos was increased to about 19.5 h compared to about 7.2 h seen in the untreated embryos. Using a deletion approach to obtain a series of mutants, Forsthoefel et al. [12] revealed that human TS molecules lacking the N-terminal 12 or 29 amino acids were quite unstable in transfected cells and had a half-life of only 1–2 h [27]. The N-terminal sequence of the zebrafish TS is different from human TS. Therefore, further study is needed to address whether the N-terminal sequence of zebrafish TS also plays some role in TS stability.

Western blot analysis confirmed that there was about a six-fold increase in TS expression in 5-FU-treated embryos compared with untreated embryos. Using a rabbit reticulocyte lysate translation system, we found that excess exogenous TS could inhibit TS mRNA translation significantly. Additionally, treatment of embryos with 5-FU increased the TS stability significantly. The results strongly support the idea that both increased TS stability and enhanced TS translation efficiency contributed to the 5-FU-induced augmentation of TS protein. Taken together, our results provide evidence that both increased TS stability as well as increased expression of TS account for the enhancement of TS protein observed in response to fluoropyrimidine treatment. These findings also suggest that the auto-regulatory mechanisms of TS may be a universal phenomenon among different species from *E. coli* to zebrafish to human beings. Additionally, our results provide evidence that some specific nucleotides and an intact stem-loop structure are necessary for the binding of TS protein to RNA. These findings enhance our understanding of the structural requirements for the interaction of RNA and protein.

## Materials and Methods

### 1. Maintenance of zebrafish embryos and drug treatment

Techniques for the caring and breeding of zebrafish have been previously described [28]. Zebrafish embryos were reared in 0.0003% PTU to prevent pigmentation and staged at 28°C. Collected embryos were maintained in an incubation buffer (15 mM NaCl, 0.5 mM KCl, 1 mM CaCl_2_, 1 mM MgSO_4_, 0.15 mM KH_2_PO_4_, 0.05 mM Na_2_HPO_4_ and 0.7 mM NaHCO_3_) at 28°C. The animal experiments were approved by the Experimental Animal Center of Shandong province, China, and the zebrafish embryos were treated in accordance with international animal ethics guidelines.

Healthy embryos at the 1–2 cell stage were dechorionated by enzymatic digestion using 1 mg/mL trypsin (Sigma, St. Louis, Missouri) for 5–10 min at room temperature. The embryos were then washed five times with the incubation buffer. Varying concentrations of 5-FU or ZD 1694 were added directly to the incubation buffer and maintained at standard condition for the specified times.

### 2. Western blot

Western blot was performed as previously described to determine the amount of TS in the developing embryos [19]. Briefly, equivalent amounts of crude protein (30 µg) from zebrafish embryos were resolved on SDS–PAGE (12% acrylamide). Gels were electro-blotted onto nitrocellulose membranes (Bio-Rad), and filter membranes were then incubated in blocking solution (1×PBS, 0.2% Tween-20 and 5% non-fat dry milk powder) for 2 h at room temperature. Membranes were incubated overnight at 4°C with the anti-TS106 monoclonal antibody (A gift from Dr. Carmen J. Allegra at the National Cancer Institute) at a dilution of 1∶2000. After four 15-min washes in PBST (1×PBS, 0.2% Tween-20), membranes were incubated with horseradish peroxidase-conjugated secondary antibodies at a dilution of 1∶2000 (IgG goat anti-mouse, Bio-Rad) for 1 h at room temperature. After an additional four 15-min washes with PBST, membranes were processed by the enhanced chemiluminescence method (SuperSignal Substrate, Pierce) and protein bands were visualized using autoradiography.

### 3. Isolation of RNA and Northern blot analysis

Zebrafish embryos were treated or untreated with 5-FU or ZD1694 in the incubation buffer and the embryos were harvested and homogenized after the specified incubation time. Total RNA was extracted using the Qiagen total RNA extraction kit (Qiagen, CA) following the manufacture's protocol. For Northern blotting analysis, 5 µg RNA was resolved on a 1% agarose/formaldehyde gel and then transferred to a positively charged BrightStar-Plus nylon membrane (Ambion, CA) by capillary transfer. Antisense RNA probes were synthesized in vitro using the MEGAshortscript T7 kit (Ambion, cat. no. 1354, CA). The TS antisense probe was derived from a PCR-generated template complementary to nucleotides 520–1216 of the zebrafish TS cDNA. RNA probes were gel-purified and biotinylated using the BrightStar Psoralen-Biotin non-isotopic labeling kit (Ambion, cat. no. 1480, CA). The membrane was pre-hybridized for 2 h at 65°C followed by probe hybridization overnight at 65°C. The BrightStar BioDetect kit (Ambion, cat. no. 1930) was then used for detection of cellular mRNAs. Quantification was performed using a Hewlett Packard ScanJet 4P and NIH image 1.51 software.

### 4. RNA gel mobility shift assay

The recombinant zebrafish TS protein was expressed using pET-28/Z-TS vector and the His-tag TS was purified using a Ni-NTA spin kit (Qiagen, CA) as previously described [19]. After purification, the His-tagged TS protein was dissolved in PBS buffer and thrombin (500 units) was added to incubate for 12 h at room temperature, and then dialyzed to PBS buffer to remove His-tag. The solution of recombinant TS protein was freeze-dried and stored at −20°C. RNA gel mobility shift experiments were performed as described previously [16]. Briefly, ^32^P-labeled full-length TS RNA transcripts (TS1–1163) were generated by in vitro transcription using the MEGAshortscript T7 kit (Ambion, cat. no. 1354). A mixture of the ^32^P-labeled TS RNA and 100 ng purified recombinant protein was incubated at 37°C for 10 min and separated on a non-denaturing polyacrylamide gel (5%, w/w, acrylamide/N,N'-methylenebisacrylamide, 60∶1). The resolved TS-RNA complex was visualized by autoradiograghy.

Competition experiments were performed as previously described [16] using 100 ng of recombinant zebrafish TS and 1 ng of ^32^P-labeled full-length TS RNA (1×10^5^ c.p.m). These conditions were selected based on experiments using a fixed amount of radiolabled TS mRNA with increasing concentrations of TS protein to determine the linearity of binding. Unlabeled TS RNAs, including full-length TS mRNA TS1–1163, TS1–580, TS1–290, TS1–145, TS1–72, TS1–36, TS13–32, TS581–1163, TS291–580, TS146–290, TS73–145, TS37–72 and luciferase mRNA at a concentration of 0–100-fold molar excess, were then added to the reaction mixture prior to addition of TS protein. The relative binding affinities (IC_50_) of TS RNA sequences for TS protein were determined in terms of the concentration of unlabeled RNA at which specific binding of ^32^P-labeled full-length TS mRNA was inhibited by 50%. Quantification was performed using a Hewlett Packard ScanJet 4P and NIH image 1.51 software. Each experiment was performed three to five times.

### 5. Immunoprecipitation and RT-PCR analysis

Immunoprecipitation and RT-PCR analysis was carried out according to our previously described methods with minor modification [7]. Briefly, zebrafish embryos were cultured in the incubation buffer for 24 h. Embryos were washed twice in ice-cold phosphate-buffered saline and lysed with NET-2 lysis buffer. The lysates were pre-cleared by incubating lysates with protein-A agarose (Invitrogen, CA) for 3 h. Samples were then transferred to new tubes for incubation with TS monoclonal antibody (1∶2000) for at least 3 h. All incubation steps were performed at 4°C. Complexes were precipitated by centrifugation at 3000×*g* and washed with washing buffer I (50 mM Tris-HCl pH 7.5, 150 mM NaCl, 1% NP-40, 0.5% sodium deoxycholate and 1 mM PMSF), high salt washing buffer II (50 mM Tris-HCl pH 7.5, 500 mM NaCl, 0.1% NP-40 and 0.05% sodium deoxycholate), low salt washing buffer III (50 mM Tris-HCl pH 7.5, 0.1% NP-40 and 0.05% sodium deoxycholate) and TE buffer (10 mM Tris pH 8.0 and 1 mM EDTA). The presence of TS protein in the precipitated complex was determined using Western blot analysis. RNA was extracted from the complex using phenol-chloroform, and precipitated by isopropyl alcohol for use as a template for cDNA synthesize using random primers (TaKaRa Bio Group, Japan) and M-MuLV reverse transcriptase (Promega, Madison, MI) according to manufacturer's protocol. The sequences of the primers used for PCR were as follows:

TS340 (sense), 5′-CGGAGAAAGCAAGAAAGAA-3′

TS1040 (antisense), 5′-GGGCGTCACCTAATGTATG-3′

PCR reactions were carried out under standard conditions (95°C for 1 min, 60°C for 1 min, 72°C for 90 s, for 30 cycles), followed by a final extension step at 72°C for 5 min. PCR products were resolved by electrophoresis using a 1.2% agarose gel stained with ethidium bromide.

### 6. TS protein stability assay for zebrafish embryos

The half-life of TS was analyzed as described previously with little modification [27]. Healthy embryos at the 1–2 cell stage were dechorionated by enzymatic digestion with 1 mg/mL trypsin (Sigma, St. Louis, Missouri) for 5–10 min at room temperature and then cultured in six-well plates with 50 embryos per well. Cycloheximide (10 µg/mL) was added to the incubation buffer. After the embryos were cultured for 2 h, 0.5 µg/mL 5-FU was added and embryos were incubated for another 4, 8, 16, 24 or 36 h. Dead and malformed embryos were removed. Untreated embryos (n = 100) and those treated with cycloheximide and 5-FU were collected for analysis of the TS level using Western blot analysis.

### 7. In vitro translation

Translation reactions were performed using a rabbit reticulocyte lysate system (Promega, Madison, MI) per the manufacturer's instructions. Briefly, the translation reaction mixture (final volume, 20 µL) containing rabbit reticulocyte lysate, an amino acid mixture without methionine (0.4 µL), ribonuclease inhibitor (20 U) and ^35^S-methionine (12 µCi) were incubated with full-length TS mRNA transcript at 30°C for 1 h. To examine the influence of exogenous TS on the translation effect of TS, various concentrations of TS were included in the translation system. The translation product was analyzed using a 12% SDS-PAGE and visualized using autoradiography after drying the gel for 1 h in a gel dryer.
